# Engagement in different sport disciplines during university years and risk of locomotive syndrome in older age: J-Fit^+ ^Study

**DOI:** 10.1186/s12199-021-00958-w

**Published:** 2021-03-22

**Authors:** Shaoshuai Shen, Koya Suzuki, Yoshimitsu Kohmura, Noriyuki Fuku, Yuki Someya, Hisashi Naito

**Affiliations:** 1 Institute of Health and Sports Science & Medicine, Juntendo University, 1-1 Hiraka-gakuendai, Inzai, Chiba, 270-1695 Japan; 2 Graduate School of Health and Sports Science, Juntendo University, 1-1 Hiraka-gakuendai, Inzai, Chiba, 270-1695 Japan; 3 Sportology Center, Graduate School of Medicine, Juntendo University, 2-1-1 Hongo, Bunkyo-ku, Tokyo, 113-8421 Japan

**Keywords:** University sports club, Sport disciplines, Locomotive syndrome, Japanese men, Historical cohort, University athletes, Middle-aged men, Older men

## Abstract

**Background:**

Among former Olympic-level athletes, engagement in different sport disciplines has been associated with mortality risk in subsequent years. However, limited evidence is available on whether engagement in different sport disciplines at a young age is associated with locomotive syndrome (LS) risk later in life. This study examined the relationship between engagement in different sport disciplines during university years and LS risk in older age among former university athletes.

**Methods:**

Participants were 274 middle-aged and 294 older men alumni who graduated from a school of physical education in Japan. LS risk was defined as answering “yes” to any of the Loco-check questions. Data on university sports club membership were collected using questionnaires. University clubs were classified into three groups of cardiovascular intensity (low, moderate, high), following the classification system of sport disciplines by the American College of Cardiology. This classification considers the static and dynamic components of an activity, which correspond to the estimated percent of maximal voluntary contraction reached and maximal oxygen uptake achieved, respectively. University clubs were grouped based on the risk of bodily collision (no, yes) and extent of physical contact (low, moderate, high). Relationships between engagement in different sport disciplines and LS risk were analyzed using Cox proportional hazards models, and adjusted for age, height, weight, joint disease, habitual exercise, and smoking and drinking status.

**Results:**

Adjusted hazard ratios and 95% confidence intervals associated with the low, moderate, and high cardiovascular intensity sports were 1.00 (reference), 0.48 (0.22–1.06, *P* = 0.070), and 0.44 (0.20–0.97, *P* = 0.042) in older men, respectively; however, there was no significant association between these parameters among middle-aged men. Engagement in sports associated with physical contact and collision did not affect LS risk in either group.

**Conclusions:**

Engagement in sports associated with high cardiovascular intensity during university years may reduce the risk of LS in later life. Encouraging young people to participate in such activities might help reduce LS prevalence among older populations.

**Supplementary Information:**

The online version contains supplementary material available at 10.1186/s12199-021-00958-w.

## Background

As the average age of the Japanese population increases, the number of older adults in need of nursing care grows annually, driving the long-term care insurance expenditure, which more than doubled from 4 to 10 trillion yen between 2000 and 2018 [1]. In this context, in 2007, the Japanese Orthopedic Association (JOA) introduced a concept of locomotive syndrome (LS) [2, 3] to describe people at high risk of musculoskeletal ambulation disability (associated with impaired mobility function, such as sit-to-stand or gait) caused by diseases of the locomotor organ [4]. This concept aims to raise awareness among people affected by or at risk of LS to maintain their health and delay or prevent the need for long-term care [5].

In Japan, LS has been identified as an independent risk factor for falls and reduced mobility in activities of daily living [6, 7], causing older adults to require long-term care [8]. In fact, LS-related complications such as fractures and falls are the fourth and musculoskeletal disorders are the fifth leading causes of the need for long-term care [9]. Meanwhile, although LS is a risk factor for older adults, its prevalence among men and women aged < 40 years is estimated at 13% and 19.4%, respectively [6]. LS among relatively young people is a cause for concern as the syndrome tends to persist in older age, driving the future long-term need for care. To develop an effective approach to prevent LS, it is necessary to understand what activities, including types of sport, performed at a young age can reduce the risk of LS.

Health benefits of physical activity and exercise are well known; governments and specialist societies worldwide recommend that people regularly participate in physical exercises [10–12]. A growing body of evidence has demonstrated the benefits of physical activity among school-aged children and adolescents [13]. For example, a study by Kidokoro et al. suggested that moderate to vigorous physical activity in this age group may reduce the adverse effects of television viewing on cardiorespiratory fitness [14]. Moreover, other studies reported that the higher levels of physical activity were associated with lower risks of cardiovascular disease [15], while endurance sports and vigorous activity could protect against coronary heart disease [16]. Furthermore, evidence suggested that increasing the level of physical exercise helped prevent type 2 diabetes mellitus [17]. Overall, physical exercise appears essential to health promotion and prevention of many diseases.

The effects of physical activity and exercise on LS risk have mostly been studied among the general population, using subjects with no regular exercise habits as the control group. Regular exercise habit in middle age has been reported to be an important factor in protection against LS in older years [18]. However, the long-term effects of different levels of intensity, bodily collision, and physical contact during exercise on the risk of LS have not been clarified. Zwiers et al. reported that engagement in high-intensity sports did not bring any survival benefits compared with low-intensity activities among former Olympic athletes [19]. The problem is that high-intensity exercise puts great strain on the body and may cause serious injuries [20]. Furthermore, it is still not elucidated whether high-intensity exercise is beneficial or increases the risk of LS among the school of physical education alumni, who have performed more physical activity and exercise during their young age, compared with the general population. Therefore, the purpose of this study was to examine the effects of physical engagement in different levels of intensity, bodily collision, and physical contact exercises during university years on LS risk in middle and older age alumni of the school of physical education. The findings will be crucial to improve disease prevention policies and education.

## Methods

### Study design and population

The J-Fit^+^ Study is a historical cohort study that investigates the association between physical fitness, motor ability, sports experience, sports club membership at a young age, and future diseases among alumni of physical education university. J-Fit^+^ Study details and participants’ characteristics have been described previously [21, 22].

**Fig. 1:**
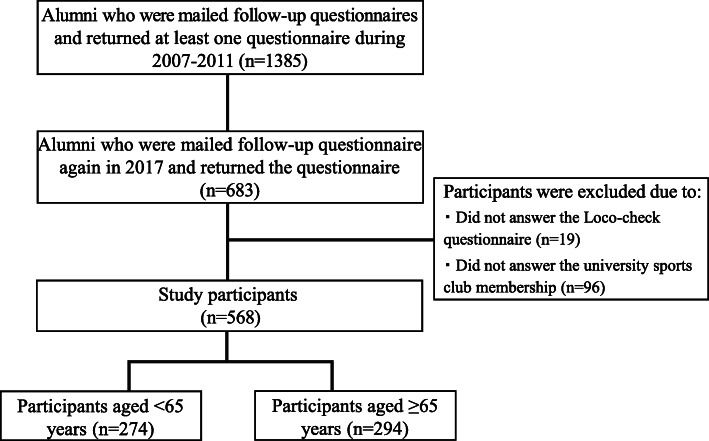
A flow-chart of participant inclusion in the present study

This study included 1385 Japanese alumni of a physical education university who had participated in follow-up investigations and responded to self-administered questionnaires at least once during 2007–2011 [23]. In 2017, of these 1385 alumni, 49.3% (*n* = 683) consented to be contacted by us and were sent a follow-up questionnaire by post regarding their height, weight, body mass index (BMI), university sports club membership, locomotive organ health (Loco-check), recent habitual exercises, joint disease, smoking status, and drinking status. Alumni who either failed to provide information on their LS risk (*n* = 19) or did not have the university sports club data (*n* = 96) were excluded. Finally, 568 individuals were included in the analysis; the samples were dichotomized by age < 65 (*n* = 274) and ≥ 65 (*n* = 294) years (Fig. 1). Ethical approval was provided by the Faculty of Health and Sports Science, Juntendo University (No. HS31–25).

### Classification of university sports club disciplines

**Fig. 2:**
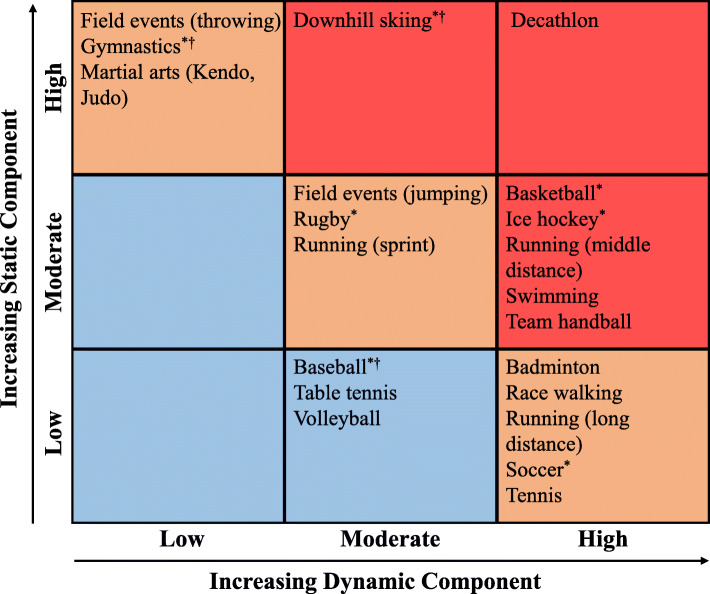
Classification of university sports club membership into three categories, low (blue), moderate (orange), and high (red) total cardiovascular intensity. The static component was defined as the estimated percent of maximal voluntary contraction reached; the dynamic component was defined as the estimated percent of maximal oxygen uptake achieved. ^*^Danger of bodily collision. ^†^Increased risk of syncope occurs [24]

The university sports club membership was assessed with the following open-ended questions: “Did you belong to a university sports club while at university? What was its name? How many weekly practice sessions did you participate in? How many hours per session?” We classified sports club membership based on the American College of Cardiology’s sports classification system [24]. This system defines three levels of cardiovascular intensity (low, moderate, high) based on the extent of dynamic or static exertion required to perform that sport during competition; these components correspond to the estimated percent of maximal oxygen uptake achieved and maximal voluntary contraction reached, respectively. In addition, total cardiovascular intensity (low, moderate, high) is the sum of static and dynamic component values. The total cardiovascular demand is shown in blue (low), orange (moderate), and red (high) in Fig. 2. This classification system also differentiates sports based on their association with a risk of bodily collision, either with another player or with an object, projectile, or the ground, as well as the degree of risk to the athletes or others if a sudden syncopal event occurs. Considering these factors, we divided sports clubs into those associated with the risk of bodily collision (including the danger of bodily collision and increased risk of syncope) and those without such risk [19]. Finally, we considered the American Academy of Pediatrics’ classification of contact sports, which accounts for the risk of injury [25]. Sports clubs were categorized into non-contact (low), limited-contact (moderate), or high-contact (high) based on the relative risk of an acute injury to the athlete.

### LS risk test (loco-check)

The following seven items were included in the Loco-check questionnaire developed by the JOA to evaluate locomotive organs: 1. You cannot put on your sock standing on one leg; 2. You often trip or slip around the house; 3. You need to hold on to the handrail when climbing the stairs; 4. You have difficulty doing moderately heavy housework; 5. You have difficulty carrying home 2 kg of shopping (e.g., equivalent to two 1-L cartons of milk); 6. You cannot walk for a quarter of an hour nonstop; 7. You cannot make it across the road before the light turns red. Participants responded to these with either “yes” or “no”. Participants who answered “yes” to one or more of these items were defined as subjects suspected of LS risk; participants who answered “no” to all seven items were defined as subjects with “no risk” of LS [26, 27].

### The assessment of lifestyle behaviors

The recent habitual exercise was measured with the following question: “Please fill in the type of sport you are currently playing (sports name), the frequency of the activities (day/week), and the time (min/day) in the table below.” The current habitual exercise was defined as the exercise performed ≥30 min at a time and ≥ 2 times per week [28]. The different types of sports played during the week were also considered as habitual exercises in this study.

Joint disease was determined with the following question: “Have you ever been diagnosed by a doctor with any joint disease?” The answers were 1 (Osteoarthritis of the knee), 2 (Herniated disc), 3 (Other 1: Disease name), and 4 (Other 2: Disease name). Participants who answered “yes” to one or more of these items were defined as subjects having joint diseases.

Smoking status was determined with the following question: “Do you smoke now?” The response options were 1 (yes), 2 (I used to smoke), and 3 (I have never smoked). Regarding drinking status, the question asked was: “Do you drink alcohol now?” The response options were 1 (yes), 2 (I used to drink), and 3 (I have never drink).

### Physical fitness and motor ability tests

Since 1973, the Department of Physical Education of our university has performed an annual test of physical fitness and motor ability of its students and stored the alumni’s data. Tests results obtained in the fourth year of university by participating alumni were used in this study. Side-step test, vertical jump, back muscle strength, grip strength, trunk lift, trunk-forward flexion, and step-test were used to assess physical fitness. In addition, 50-m run, 1500-m run, running long jump, hand-ball throw, and pull-up were used as motor ability tests. Details of these tests are presented in our previous report [29].

### Statistical analysis

Statistical analyses were performed with IBM SPSS Statistics for Windows, version 21.0 (SPSS Inc., Chicago, IL, USA), and the level of significance was set at *p*-value < 0.05. Continuous variables are expressed as medians (interquartile range). Categorical variables are presented as numbers (percentage). Cox proportional hazards model was used to examine the association between sports club membership during university and LS risk in later life. In this study, the time of the follow-up survey was regarded as the time during which they were suspected of LS risk. The time at which an alumnus graduated and at which the follow-up survey was conducted were considered the beginning and the end of the follow-up period. The following models were constructed in Cox proportional hazards analysis. Model 1 was adjusted for age, height, weight, joint disease, habitual exercise, and smoking and drinking status. Model 2 was additionally adjusted for cardiovascular intensity, bodily collision, and physical contact. Independent sample t-tests were used to examine differences in physical fitness and motor ability test results obtained at university from alumni included in the low vs. high total cardiovascular intensity group.

## Results

Overall, 568 participants were included in this study, including 274 middle-aged and 294 older men. Table 1 summarizes the characteristics of all participants, as well as of middle-aged and older men. Among middle-aged men, during the follow-up period, LS risk was suspected in 6 (13.6%), 23 (13.8%), and 11 (17.5%) participants performing low, moderate, and high total cardiovascular intensity exercises, respectively (Table 1). In older men, during the follow-up period, LS risk was suspected in 31 (53.4%), 62 (34.6%), and 21 (36.8%) participants performing low, moderate, and high total cardiovascular intensity exercises, respectively (Table 1).

**Table 1:** Characteristics of all participants and by different levels of cardiovascular

	All (*n* = 568)			Middle-aged (*n* = 274)			Elderly (*n* = 294)		
	Low cardiovascular intensity	Moderate cardiovascular intensity	High cardiovascular intensity	Low cardiovascular intensity	Moderate cardiovascular intensity	High cardiovascular intensity	Low cardiovascular intensity	Moderate cardiovascular intensity	High cardiovascular intensity
Number of participants	102	346	120	44	167	63	58	179	57
Age^a^ (years)	66.0 (58.8, 75.0)	65.0 (57.0, 71.0)	64.0 (57.0, 69.0)	57.0 (54.3, 63.0)	57.0 (53.0, 61.0)	57.0 (54.0, 61.0)	73.0 (69.0, 78.3)	71.0 (68.0, 74.0)	69.0 (67.5, 74)
Height^a^ (cm)	170.0 (167.0, 176.9)	170.0 (165.0, 174.0)	172.0 (167.0, 177.0)	171.1 (167.1, 179.5)	172.0 (168.0, 176.0)	174.0 (171.0, 178.0)	169.3 (166.0, 173.5)	168.0 (163.0, 172.0)	168.0 (165.0, 173.8)
Weight^a^ (kg)	68.5 (64.0, 77.1)	68.0 (62.0, 75.0)	70.0 (65.0, 75.8)	68.5 (66.0, 79.2)	72.0 (65.0, 76.5)	71.0 (68.0, 80.0)	68.0 (63.0, 76.0)	65.4 (60.0, 72.0)	69.0 (62.5, 74.5)
BMI^a^ (kg/m^2^)	23.8 (22.3, 25.8)	23.7 (22.0, 25.3)	23.9 (22.3, 25.6)	23.8 (22.2, 26.0)	23.7 (22.2, 25.4)	23.7 (22.1, 25.8)	23.8 (22.3, 25.6)	23.5 (21.8, 25.2)	24.2 (22.5, 25.5)
Training frequency at university ^a,c^ (day/week)	6.0 (6.0, 7.0)	6.0 (6.0, 6.0)	6.0 (6.0, 6.0)	6.0 (6.0, 7.0)	6.0 (6.0, 7.0)	6.0 (6.0, 6.0)	6.0 (6.0, 7.0)	6.0 (6.0, 6.0)	6.0 (6.0, 6.5)
Training time at university ^a,c^ (min/day)	180.0 (150.0, 180.0)	150.0 (120.0, 180.0)	150.0 (120.0, 180.0)	180.0 (135.0, 180.0)	150.0 (120.0, 180.0)	180.0 (120.0, 180.0)	180.0 (150.0, 180.0)	150.0 (120.0, 180.0)	150.0 (120.0, 180.0)
Follow-up period^a^ (years)	44.5 (36.8, 52.0)	43.0 (35.0, 48.3)	42.0 (35.0, 47.0)	35.0 (32.3, 40.0)	35.0 (31.0, 39.0)	35.0 (32.0, 39.0)	51.0 (46.8, 56.0)	48.0 (45.0, 52.0)	47.0 (45.0, 52.5)
Follow-up (person-years)	4533	14,576	4999	1570	5804	2224	2963	8772	2775
Locomotive syndrome risk^a^ (n,%)	37(36.3)	85(24.6)	32(26.7)	6(13.6)	23(13.8)	11(17.5)	31(53.4)	62(34.6)	21(36.8)
Joint disease^a^ (n,%)	24(23.5)	81(23.4)	30(25.0)	10(22.7)	37(22.2)	10(15.9)	14(24.1)	44(24.6)	20(35.1)
Habitual exercise, yes^a^ (n, %)	53(52.0)	210(60.7)	63(52.5)	23(52.3)	98(58.7)	33(52.4)	30(51.7)	112(62.6)	30(52.6)
Smoking status^a^ (n, %)									
Never smoker	20 (19.6)	142 (41.0)	26 (21.7)	10 (22.7)	84 (50.3)	16 (25.4)	10 (17.2)	58 (32.4)	10 (17.5)
Current smoker	19 (18.6)	43 (12.4)	17 (14.2)	13 (29.5)	19 (11.4)	8 (12.7)	6 (10.3)	24 (13.4)	9 (15.8)
Former smoker	63 (61.8)	161 (46.5)	77 (64.2)	21 (47.7)	64 (38.3)	39 (61.9)	42 (72.4)	97 (54.2)	38 (66.7)
Drinking status^a^ (n, %)									
None	12 (11.8)	41 (11.8)	12 (10.0)	1 (2.3)	15 (9.0)	4 (6.3)	11 (19.0)	26 (14.5)	8 (14.0)
Current	83 (81.4)	275 (79.5)	98 (81.7)	40 (90.9)	142 (85.0)	55 (87.3)	43 (74.1)	133 (74.3)	43 (75.4)
Former	7 (6.9)	30 (8.7)	10 (8.3)	3 (6.8)	10 (6.0)	4 (6.3)	4 (6.9)	20 (11.2)	6 (10.5)
Age^b^ (years)	21.0 (21.0, 22.0)	21.0 (21.0, 22.0)	21.0 (21.0, 22.0)	21.0 (21.0, 22.0)	21.0 (21.0, 21.0)	21.0 (21.0, 22.0)	21.0 (21.0, 22.0)	21.0 (21.0, 22.0)	21.0 (21.0, 22.0)
Year of graduation^b^	1973 (1965, 1980)	1974 (1969, 1982)	1975 (1970, 1982)	1982 (1977, 1984)	1982 (1978, 1986)	1982 (1978, 1985)	1966 (1961, 1970)	1969 (1965, 1972)	1970 (1965, 1972)

The data are presented as medians (interquartile range) for continuous variables and number (percentage) for categorical variables

BMI, body mass index

^a^ Data collected at the time of follow-up questionnaire submission

^b^ Data collected at the time participant attended college

^c^ In all, Low cardiovascular intensity group *n* = 93, Moderate cardiovascular intensity group *n* = 324, High cardiovascular intensity group *n* = 112 In middle-aged, Low cardiovascular intensity group *n* = 41, Moderate cardiovascular intensity group *n* = 157, High cardiovascular intensity group *n* = 59 In elderly, Low cardiovascular intensity group *n* = 52, Moderate cardiovascular intensity group *n* = 167, High cardiovascular intensity group *n* = 53

Table 2 demonstrates the university sports club disciplines stratified by three levels of total cardiovascular intensity (low, moderate, high). The number of volleyball club members in the low cardiovascular intensity exercise group was 61 (59.8%). The number of gymnastics club members in the moderate cardiovascular intensity exercise group was 65 (18.8%). The number of basketball club members in high cardiovascular intensity exercise group was 61 (50.8%) (Table 2).

**Table 2:** College sports club disciplines, stratified by three levels of cardiovascular intensity (low, moderate, and high)

	All(*n* = 568)	Middle-aged(*n* = 274)	Elderly(*n* = 294)
Low cardiovascular intensity			
Number of participants	102	44	58
Volleyball	61 (59.8)	22 (50.0)	39 (67.2)
Baseball	37 (36.3)	19 (43.2)	18 (31.0)
Table tennis	4 (3.9)	3 (6.8)	1 (1.7)
Moderate cardiovascular intensity			
Number of participants	346	167	179
Gymnastics	65 (18.8)	19 (11.4)	46 (25.7)
Soccer	46 (13.3)	32 (19.2)	14 (7.8)
Running (sprint)	45 (13.0)	28 (16.8)	17 (9.5)
Field events (jumping)	43 (12.4)	21 (12.6)	22 (12.3)
Running (long distance)	43 (12.4)	17 (10.2)	26 (14.5)
Field events (throwing)	35 (10.1)	12 (7.2)	23 (12.8)
Tennis	31 (9.0)	13 (7.8)	18 (10.1)
Martial arts (Kendo, Judo)	30 (8.7)	19 (11.4)	11 (6.1)
Rugby	6 (1.7)	6 (3.6)	0 (0.0)
Badminton	1 (0.3)	0 (0.0)	1 (0.6)
Race walking	1 (0.3)	0 (0.0)	1 (0.6)
High Cardiovascular intensity			
Number of participants	120	63	57
Basketball	61 (50.8)	32 (50.8)	29 (50.9)
Team handball	21 (17.5)	10 (15.9)	11 (19.3)
Running (middle distance)	14 (11.7)	6 (9.5)	8 (14.0)
Downhill skiing	10 (8.3)	3 (4.8)	7 (12.3)
Decathlon	7 (5.8)	7 (11.1)	0 (0.0)
Swimming	6 (5.0)	4 (6.3)	2 (3.5)
Ice hockey	1 (0.8)	1 (1.6)	0 (0.0)

The data are presented as number (percentage)

In Model 1, there was no association between any sports category and the risk of LS in all age and in either middle-aged or older men (Tables 3, 4 and 5). In Model 2, neither risk of bodily collision nor physical contact was associated with the risk of LS in all age and in either middle-aged or older men (Tables 3, 4 and 5). However, in Model 2, engagement in exercise of increased cardiovascular intensity was associated with a lower risk of LS; hazard ratio for moderate-intensity exercise was 0.48 (95% confidence interval [CI]: 0.22–1.06; *P* = 0.070) and that for high-intensity exercise was 0.44 (95% CI: 0.20–0.97; *P* = 0.042) among older men (Table 5).

**Table 3:** Hazard ratios associated with locomotive syndrome according to different intensity of exercise and risk of bodily collision and physical contact based on all sample

		LS-risk group	Model 1		Model 2	
		n (%)	HR (95% CI)	*P-*value	HR (95% CI)	*P-*value
All (n = 568)						
Intensity						
Cardiovascular						
Low	102	37 (36.3)	1.00 (Reference)		1.00 (Reference)	
Moderate	346	85 (24.6)	0.82 (0.53 to 1.25)	0.350	0.63 (0.33 to 1.21)	0.166
High	120	32 (26.7)	0.87 (0.53 to 1.43)	0.587	0.77 (0.40 to 1.47)	0.424
Sport type						
Bodily collision						
No	312	90 (28.8)	1.00 (Reference)		1.00 (Reference)	
Yes	256	64 (25.0)	0.80 (0.56 to 1.13)	0.201	0.84 (0.41 to 1.76)	0.651
Physical contact						
Low	180	48 (26.7)	1.00 (Reference)		1.00 (Reference)	
Moderate	170	53 (31.2)	0.86 (0.57 to 1.30)	0.464	0.67 (0.38 to 1.18)	0.163
High	218	53 (24.3)	0.72 (0.47 to 1.10)	0.128	0.82 (0.35 to 1.93)	0.648

*HR* hazard ratio, *CI* confidence interval

The data are presented as hazard ratio (95% confidence interval)

Model 1 adjusted for age, height, weight, joint disease, habitual exercise, smoking status, and drinking status

Model 2 additionally adjusted for cardiovascular intensity of exercise and risk of bodily collision and physical contact

**Table 4:** Hazard ratios associated with locomotive syndrome according to different intensity of exercise and risk of bodily collision and physical contact based on middle-aged sample

		LS-risk group	Model 1		Model 2	
		n (%)	HR (95% CI)	*P-*value	HR (95% CI)	*P-*value
Middle-aged (n = 274)						
Intensity						
Cardiovascular						
Low	44	6 (13.6)	1.00 (Reference)		1.00 (Reference)	
Moderate	167	23 (13.8)	1.03 (0.40 to 2.63)	0.950	1.42 (0.36 to 5.58)	0.619
High	63	11 (17.5)	1.62 (0.59 to 4.49)	0.350	2.32 (0.60 to 8.90)	0.220
Sport type						
Bodily collision						
No	143	22 (15.4)	1.00 (Reference)		1.00 (Reference)	
Yes	131	18 (13.7)	0.70 (0.35 to 1.41)	0.319	1.17 (0.23 to 5.90)	0.847
Physical contact						
Low	83	12 (14.5)	1.00 (Reference)		1.00 (Reference)	
Moderate	80	13 (16.3)	1.00 (0.45 to 2.24)	0.991	1.05 (0.38 to 2.85)	0.929
High	111	15 (13.5)	0.70 (0.30 to 1.59)	0.391	0.52 (0.08 to 3.25)	0.483

*HR* hazard ratio, *CI* confidence interval

The data are presented as hazard ratio (95% confidence interval)

Model 1 adjusted for age, height, weight, joint disease, and habitual exercise

Model 2 additionally adjusted for cardiovascular intensity of exercise and risk of bodily collision and physical contact

**Table 5:** Hazard ratios associated with locomotive syndrome according to different intensity of exercise and risk of bodily collision and physical contact based on elderly sample

		LS-risk group	Model 1		Model 2	
		n (%)	HR (95% CI)	*P-*value	HR (95% CI)	*P-*value
Elderly (n = 294)						
Intensity						
Cardiovascular						
Low	58	31 (53.4)	1.00 (Reference)		1.00 (Reference)	
Moderate	179	62 (34.6)	0.83 (0.51 to 1.35)	0.442	0.48 (0.22 to 1.06)	0.070
High	57	21 (36.8)	0.67 (0.37 to 1.22)	0.187	0.44 (0.20 to 0.97)	0.042
Sport type						
Bodily collision						
No	169	68 (40.2)	1.00 (Reference)		1.00 (Reference)	
Yes	125	46 (36.8)	0.94 (0.63 to 1.39)	0.756	0.72 (0.30 to 1.73)	0.468
Physical contact						
Low	97	36 (37.1)	1.00 (Reference)		1.00 (Reference)	
Moderate	90	40 (44.4)	0.85 (0.52 to 1.39)	0.516	0.57 (0.28 to 1.17)	0.126
High	107	38 (35.5)	0.88 (0.54 to 1.42)	0.595	1.25 (0.45 to 3.46)	0.672

*HR* hazard ratio, *CI* confidence interval

The data are presented as hazard ratio (95% confidence interval)

Model 1 adjusted for age, height, weight, joint disease, habitual exercise, smoking status, and drinking status

Model 2 additionally adjusted for cardiovascular intensity of exercise and risk of bodily collision and physical contact

## Discussion

The present study has shown that engagement in sports with a risk of collision or bodily contact during university sports did not impact LS risk in older age. In contrast, engagement in high cardiovascular intensity sports during the same period was associated with a significantly lower risk of LS among older men. These findings suggest that engaging in sports of high cardiovascular intensity at a younger age may help reduce the risk of LS in late adulthood.

In the present study, men aged ≥65 years were more likely to be affected by LS (low: 53.4%, moderate: 34.6%, high: 36.8%) than men aged < 65 years (low: 13.6%, moderate: 13.8%, high: 17.5%); these estimates are consistent with those previously reported [30]. Concurrently, this study suggests that engagement in high-intensity sports during university does not reduce the risk of LS in middle-aged men compared to engagement in low-intensity sports. Tanimoto et al. [31] reported that the total body muscle mass decreased in Japanese men around the age of 40 years and then at the age of 65 years and beyond, where it tended to decrease rapidly. Kortebein et al. [32] reported that older people tended to lose lean leg mass faster than young people. In addition, Goodpaster et al. [33] reported that age-associated loss of muscle mass was a major factor in age-associated muscle strength decline. Altogether, these findings suggest that the decline in physical strength is slower among middle-aged than among older adults. Compared with older adults, middle-aged adults tend to maintain relatively high levels of physical function. This discrepancy might account for the differences in the impact of high-intensity exercise during university years on the risk of LS in older age, which, in contrast to older adults, was not observed among middle-aged men. In this study, older men with a history of high-intensity exercise during university years were at a lower risk of LS than their counterparts with a history of low-intensity sports.

Engagement in high cardiovascular intensity sports during university years reduced the risk of LS in older men. Specifically, playing basketball or team handball, and practicing middle-distance running, among others, were associated with a lower LS risk compared to playing volleyball, baseball, or table tennis. These results are similar to those of previous studies. For example, Lemez and Baker [34] reported that, compared with the general population, elite athletes had longer life expectancy. In addition, Sarna et al. [35] reported that among world-class male athletes, those engaged in endurance and team sports had lower premature mortality rates. Teramoto and Bungum [36] speculated about the reasons for these findings; they proposed that participating in higher volumes of exercise training would lead to higher physical fitness levels, and elite athletes are generally the healthiest individuals. Our findings on the differences between low and high cardiovascular intensity exercise groups in physical fitness and motor ability test results during university years support this interpretation; specifically, participants engaged in high-intensity sports performed better on 1500-m runs and running long jump tests than those engaged in low-intensity sports (Additional file 1 Supplementary Table 1). These findings suggest that people who are engaged in high cardiovascular intensity sports during university years have higher physical fitness levels than those who are engaged in low cardiovascular intensity sports. We speculate that these relatively higher physical fitness levels are maintained in older age, reducing the risk of LS. Our findings are in contrast to those of a previous study that showed no association between engagement in high cardiovascular intensity sports and premature mortality risk in later life among former Olympic athletes [19]. However, it is plausible that this kind of activity does not affect premature mortality risk while concurrently reducing the risk of LS.

However, the risk of LS was not significantly associated with engagement in high cardiovascular intensity sports during university years in Model 1. One of the potential explanations may be the influence of risk of collision and bodily contact as confounding factors, which were not considered in Model 1. Previous studies have reported that the risks of collision and bodily contact were associated with injuries [20, 37], suggesting their confounding effects on the results in the present study. Therefore, in Model 2, we identified a negative relationship between engagement in high cardiovascular intensity sports during university years and the risk of LS when the risks of collision and bodily contact were considered.

Collision risk and physical contact associated with different university sports did not affect the risk of LS in later life in the present study. However, higher mortality risk has been associated with disciplines involving high levels of physical contact among former Olympic athletes [19] who experience a significant burden of injuries associated with their chosen disciplines [20, 37]. These injuries may gradually accumulate and have long-term effects [19]. The possible explanations for the discrepancy in findings between the present and previous studies are as follows. The previous studies involved former Olympic athletes, whose training frequency and intensity were likely higher than those of the present study participants; as a result, the cumulative extent of sustained injuries as well as the rate of physical impact were likely greater among the former than among the latter group. Although we have not found evidence that contact sports played during university years increase the risk of LS in later life, avoiding these sports reduces the risk of injuries and might thus be beneficial for overall health; using protective gear while playing these sports might also help prevent injury.

This study has several limitations, which should be considered when interpreting its findings. First, since the department of physical education of the university did not enroll female students before 1991, our survey included only men. Future studies should examine these associations in women. In addition, we did not include a control group that did not engage in any sports club during university years. Therefore, further studies should clarify the association between engagement in low to moderate cardiovascular intensity sports during university years and the risk of LS in later life compared with a matched control group. Second, this study involved alumni of a physical education department at a university; whether the presented findings generalize to a broader population is not clear. Third, we evaluated sports club membership during university years and LS risk in older age using a self-report questionnaire, which may lead to misclassification. Furthermore, our study participants were alumni who responded to a follow-up questionnaire; therefore, selection bias was possible in the present study. The study participants might be more health-conscious than the non-respondents. Thus, among still healthy middle-aged men, only 6 participants who engaged in low cardiovascular intensity sports during university years were at the risk of developing LS. This small number resulted in an increased risk of LS with the increased exercise of cardiovascular intensity. Therefore, future studies based on middle-aged men data are required to clarify this relationship. Fourth, the time of the follow-up survey was regarded as the time during which they were suspected of LS risk in this study, therefore there is a possibility that the subject had already suspected of LS risk before then. Fifth, at least ten events (the subjects who suspected of LS risk) needed for each included explanatory variable in the model [38]. However, the subjects who suspected of LS risk were only 40 in middle-aged men (Table 2), further studies with a larger sample size are required to show stable resutls. Finally, our study did not consider changes to levels and type of sports practiced during the follow-up period. However, when examining the relationship between sports engagement during university years and the risk of LS in later life, we adjusted our estimates for present exercise habits.

## Conclusions

We conducted a historical cohort survey to investigate the relationship between engagement in different sport disciplines during university and LS risk in later life. Our study demonstrated an association between engagement in a high level of cardiovascular intensity sports during university years and the risk of LS in later life. Our findings suggest that engagement in high cardiovascular intensity sports during university years may reduce the risk of LS in later life.

## Supplementary Information

**Additional file 1: Supplementary Table 1.** Comparison of physical fitness and motor ability test results during university years between low and high cardiovascular intensity groups.

## Data Availability

The datasets analyzed in the current study are available from the corresponding authors on reasonable request.

## Abbreviations

BMI: Body mass index
CI: Confidence interval
HR: Hazard ratios
JOA: Japanese Orthopedic Association
LS: Locomotive syndrome
