# Supplementary material for: Engagement in different sport disciplines during university years and risk of locomotive syndrome in older age: J-Fit+ Study
Source: Environ Health Prev Med. 2021 Mar 22;26:36. doi: 10.1186/s12199-021-00958-w (PMC7983270; doi:10.1186/s12199-021-00958-w)
Supplement: Supplementary file 1 — Additional file 1: Supplementary Table 1. Comparison of physical fitness and motor ability test results during university years between low and high cardiovascular intensity groups. [file 12199_2021_958_MOESM1_ESM.docx]

**Additional file 1**

Supplementary Table 1 Comparison of physical fitness and motor ability test results during university years between low and high cardiovascular intensity groups

|  | Low cardiovascular  intensity group | | High cardiovascular  intensity group | | t-test *P-*value^a^ |
| --- | --- | --- | --- | --- | --- |
|  |  |  |  |  |  |
| Number of participants, all | 31 | | 53 | |  |
| Physical fitness tests |  |  |  |  |  |
| Side-step test, point | 52.0 | (3.1) | 52.7 | (4.0) | 0.432 |
| Vertical jump test, cm | 67.5 | (6.2) | 64.8 | (5.7) | 0.052 |
| Back muscle strength, kg | 160.3 | (18.6) | 168.6 | (22.1) | 0.083 |
| Grip strength, kg | 51.0 | (4.4) | 50.8 | (5.2) | 0.818 |
| Trunk lift, cm | 60.9 | (6.3) | 59.0 | (7.5) | 0.233 |
| Trunk-forward flexion, cm | 14.3 | (5.0) | 13.3 | (4.9) | 0.393 |
| Step-test^b^ | 68.3 | (13.0) | 74.1 | (15.9) | 0.088 |
| Motor ability tests |  |  |  |  |  |
| 50-m run, s | 7.1 | (0.3) | 7.1 | (0.3) | 0.675 |
| 1500-m run, s | 342.0 | (29.5) | 327.6 | (29.9) | 0.036 |
| Running long jump, cm | 519.5 | (30.2) | 535.3 | (33.9) | 0.035 |
| Hand-ball throw^c^, m | 31.9 | (3.6) | 32.0 | (4.0) | 0.848 |
| Pull-up, point | 12.2 | (4.6) | 12.0 | (4.2) | 0.845 |

The data are presented as mean (standard deviation)

^a^ *P-*value of independent-samples t-test

^b^ Step-test is scored by the index derived from the formula shown in our previous report

^c^ High cardiovascular intensity group n=52
